# Cryo2Struct : A Large Labeled Cryo-EM Density Map Dataset for AI-based Reconstruction of Protein Structures

**DOI:** 10.1101/2023.06.14.545024

**Published:** 2023-06-15

**Authors:** Nabin Giri, Jianlin Cheng

**Affiliations:** Department of Electrical Engineering and Computer Science, NextGen Precision Health Institute, University of Missouri, Columbia, MO 65211, USA

## Abstract

The advent of single-particle cryogenic electron microscopy (cryo-EM) has brought forth a new era of structural biology, enabling the routine determination of large biological protein complexes and assemblies at atomic resolution. The high-resolution structures of protein complexes and assemblies significantly expedite biomedical research and drug discovery. However, automatically and accurately reconstructing protein structures from high-resolution density maps generated by cryo-EM is still time-consuming and challenging when template structures for the protein chains in a target protein complex are unavailable. Artificial intelligence (AI) methods such as deep learning trained on limited amounts of labeled cryo-EM density maps generate unstable reconstructions. To address this issue, we created a dataset called Cryo2Struct consisting of 7,600 preprocessed cryo-EM density maps whose voxels are labelled according to their corresponding known protein structures for training and testing AI methods to infer protein structures from density maps. It is larger and has better quality than any existing, publicly available dataset. We trained and tested deep learning models on Cryo2Struct to make sure it is ready for the large-scale development of AI methods for reconstructing protein structures from cryo-EM density maps. The source code, data and instructions to reproduce our results are freely available at https://github.com/BioinfoMachineLearning/cryo2struct.

## 1 Introduction

Accurately determining three-dimensional (3D) structure of proteins is critical for unlocking key insights into their molecular functions and interactions with other proteins as well as small molecules like ions, ligands, and therapeutic drugs [1]. In recent years, cryo-EM [2] has emerged as the most important technology for experimentally determining the structures of large protein complexes and assemblies that are difficult or impossible for other experimental techniques such as X-ray crystallography or Nuclear Magnetic Resonance (NMR) to solve or for cutting-edge protein structure prediction methods such as AlphaFold2 [3] to predict. The field of cryo-EM is advancing at a rapid pace with improvements in image data collection and processing techniques [4] . This progress has resulted in large amounts of high-resolution cryo-EM images of protein complexes and assemblies, and 3D density maps constructed from the 2D images to capture the 3D shapes (e.g., positions of atoms in 3D space) of proteins.

However, reconstructing atomic 3D structures of large protein complexes from 3D cryo-EM density maps is still a very challenging problem that often requires extensive manual intervention. The task is particularly difficult if the structures of the individual chains (units) in large protein complexes are not available or cannot be predicted from sequences by cutting-edge protein structure prediction methods such as AlphaFold2 [3] which serve as templates to fit into cryo-EM density maps for building the structures of the complexes.

To address this problem, significant efforts have been put into developing machine learning, particularly deep learning (DL) methods [5], to directly reconstruct structures or structural features from 3D cryo-EM density maps as the electron density in the voxels (cells) in the high-resolution density maps contain sufficient information about protein structures in most cases. Therefore, sophisticated deep learning models trained with 3D density maps as input and their corresponding structures or structural features as labels can potentially infer protein structures that fit the cryo-EM density maps accurately [6].

However, designing and implementing deep learning-based methods for reconstructing protein structures from cryo-EM density maps requires a large, standard, labeled cryo-EM dataset. Different methods have been trained previously to predict different aspects of protein structures from cryo-EM density maps, nevertheless, up to now, there is still no work on generating a publicly available, large, labeled dataset to push the frontier of the structural reconstruction from cryo-EM density maps. One reason for this bottleneck was on the scarcity of high-resolution cryo-EM density maps being deposited in Electron Microscopy Data Bank (EMDB) [7] in the past. Recently, the new cryo-EM technological advancement has resulted in a surge of high-resolution cryo-EM density maps being deposited in the EMDB [7] and their corresponding protein structures in the Protein Data Bank (PDB) [8]. Leveraging this precious resource, we created cryo2struct, a large labeled cryo-EM density map dataset for developing and testing AI-based methods to predict atomic protein structures from cryo-EM density maps. We also trained and tested deep learning models on the dataset to rigorously validate its quality. Cryo2Struct dataset is the first, large, publicly available cryo-EM dataset with standardized input features and well-curated output labels that is fully ready for the AI and machine learning development in the field.

## 2 Related Work

### 2.1 Protein Structure Reconstruction from Simulated Density Maps

Early methods for predicting structures from cryo-EM density maps utilized protein structures in the PDB to generate theoretical density maps at different resolution, usually referred to as simulated density maps for training and testing. For instance, Cascaded-CNN [9] utilized pdb2mrc from the EMAN2 package [10], and VESPER [11] utilized pdb2vol from the Situs package [12] to generate simulated density maps. However, simulated density maps lack the complexity of real-world density maps such as high noise, missing density values, and experimental artifacts that arise from protein particle picking [13] errors, electron beam interaction with atoms, or atom movement during image capture in the cryo-EM data collection and preprocessing. As such, deep learning models trained on simulated density maps may not perform well on noisy experimental density maps. Therefore, real-world cryo-EM density maps with labels need to be created to further advance the field.

### 2.2 Protein Structure Reconstruction from Experimental Density Maps

As more and more experimentally determined density maps became readily available in the EMDB, a recent method DeepTracer [14] used 1,800 experimental maps to predict the positions and amino acid types of C*α* atoms of proteins, which were then aligned with protein sequences to build protein structures. Haruspex [15] used 239 experimental maps for training and an independent test set of 122 experimental maps for testing, and EMNUSS [16] used 120 and 43 experimental density maps for training and testing respectively. ModelAngelo [17] used 3,715 experimental maps for training and a test set of 177 maps for testing. CR-I-TASSER[18], EMNUSS [16], and Emap2sec [19] employed a hybrid approach that combined both simulated maps and experimental maps in their training and validation processes. Despite the significant progress, the datasets used in these works only account for a small portion of high-resolution density maps available in the EMDB and they are not publicly available for the AI and machine learning community to use. Significantly, Giri et al. [5] highlight the criticality of developing high-quality cryo-EM datasets specifically for the training and evaluation of deep learning methods.

This work leverages the large amounts of cryo-EM density maps in the EMDB and extends the previous works by making the following contributions:

- We created the *first* large, publicly available, standardized dataset containing 7,600 labeled density maps.
- We rigorously validated the quality of the dataset by applying deep transformer models to predict the positions of key backbone atoms (i.e., C*α* atoms) of proteins and their amino acid types from cryo-EM density maps.
- We developed the *first* Hidden Markov Model (HMM) for aligning protein sequences with C*α* atoms predicted from density maps to build protein structures.
- The dataset is well formatted and documented to enable AI and machine learning experts who have no prior experience in cryo-EM data analysis to develop AI and machine learning methods to reconstruct protein structures from density maps, greatly lowering the barrier to enter into this important field.

## 3 Dataset Composition

Cryo2Struct dataset contains the necessary metadata, 3D density map files, and labels to enable AI experts without much domain knowledge to develop AI methods for reconstruction of protein structures from cryo-EM density maps.

The following data files for each cryoEM density map are provided in its individual directory:

- The original cryo-EM density map with its corresponding protein structure in the PDB format, the original protein sequence in the FASTA format [20], and the sequence extracted from the PDB structure for the residues whose positions have been determined in the structure.
- The alignment between the original sequence and the sequence extracted from the protein structure generated by Clustal Omega [21]. The two sequences are usually highly similar but not identical because some residues in the original sequence may be disordered and do not have positions (i.e, x, y, z coordinates) in the protein structure.
- The resampled and normalized cryo-EM density map generated from the original density map. The values of the voxels in this density map are normalized into the range [0, 1] from their values in the original density map and are ready for being used as input for AI and machine learning models.
- The labeled mask density maps in which the voxels containing the key backbone atoms (carbon-alpha (C*α*), nitrogen (N) and another carbon (C)) of residues (amino acids), the C*α* atoms only, the twenty different types of amino acids that C*α* atoms belong to, and the three different types of secondary structures (helix, strand, coil) of the C*α* atoms are labeled. The labels can be used as targeted outputs for AI and machine learning models to predict from the resampled and normalized density maps (input).

## 4 Data Preparation

**Figure 1:**
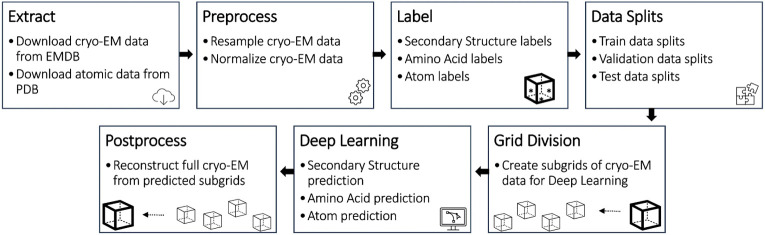
The data preparation and evaluation pipeline for Cryo2Struct dataset.

The Cryo2Struct dataset was prepared by a data processing pipeline shown in Figure 1. The data generated from each stage of the pipeline were verified to ensure the details of original experimental density maps were well preserved. The source code of the data preparation pipeline is released at the GitHub repository of Cryo2Struct for users to reproduce the process. The details of each data preparation stage are described in the following subsections.

### 4.1 Data Extraction

Cryo2Struct was curated from the experimental cryo-EM density maps released till 27 March 2023. We downloaded relatively high-resolution cryo-EM density maps for single-particle proteins with a resolution between 1 and 4 Angstrom (Å). In total 9,500 cryo-EM density maps were collected from the EMDB [7]. Similarly, we downloaded the atomic protein structures corresponding to the density maps from the PDB [8]. The downloaded PDB structures were used to label the voxels of the density maps.

We further filtered out the density maps that do not have structures in the PDB. After the filtering, 7,600 cryo-EM density maps were left for further processing. A supplementary document containing the meta information such as the corresponding PDB entry for each density map, resolution of the density map, structure determination method, software used to determine the density map, the title and the journal of the article describing the density maps is provided in the supplementary materials.

### 4.2 Preprocessing

The experimental cryo-EM density maps are determined by using cryogenic electron microscopy devices to project electrons on the top of vitrified aqueous protein samples in vacuum. The protein particle images capturing electron scattering are generated and used to build the 3D density maps of the protein. The amount of electron scattered are recorded and saved as a density map file in .MRC [22] format. The .MRC file format contains the recorded data in a 3-dimensional (3D) numpy array, often referred to as 3D grid. Each voxel also known as pixel in a 3D density map contains a value indicating if an atom of the protein may be present in the voxel. We refer to these density maps as original raw density maps, which need to be further preprocessed so they can be used to train machine learning and AI methods. Building upon established practices and techniques [9, 14], the preprocessing is implemented in two steps: (a) density map resampling and (b) density map normalization as follows.

#### Resample cryo-EM density maps:

**Figure 2:**
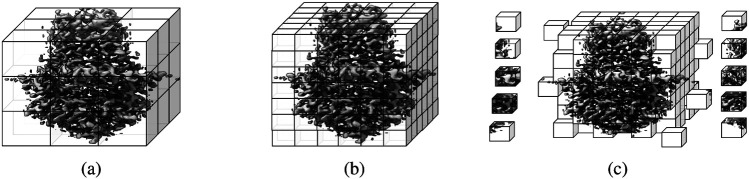
An example of density map grid resampling and division of a cryo-EM density map. (**a**) Density map (EMD-22898) in the original grid. (**b**) Resampled density map to the uniform grid size of 1Å. (**c**) Grid division of the density map.

Different raw cryo-EM density maps which are saved in a 3D grid usually have different voxel sizes, which need to be standardized. We resampled the density maps to an uniform voxel grid of 1 Å, using *vol resample* command within UCSF ChimeraX [23] in the non-interactive mode. The idea of resampling density map is illustrated in Figure 2a and 2b. The resampled density map has uniform spacing between grid points and are used for the following normalization step.

#### Normalize cryo-EM density maps:

We applied scaling and clipping to normalize the density values of the resampled cryo-EM density maps. In the cryo-EM density maps, non-zero density values represent regions (voxels) where the protein is likely present. The range of these values can differ from maps to maps with some maps containing values in one range (e.g., [−2.32, 3.91]) and others in another range (e.g., [−0.553, 0.762]). To make the density values of different density maps comparable, we perform the percentile normalization by first calculating 95^*th*^ percentile of the non-zero density values in a density map and then dividing the values in the map by this value to scale the map such that the highest density value is 1. To deal with the extreme outliers in the density values, we set all values below 0 to 0 and all values above 1 to 1 after the division. The normalization removes the cross-map difference caused by differences in experimental conditions, sample preparation, and software used to process the maps, allowing machine learning and AI methods to learn patterns across different cryo-EM maps.

### 4.3 Labeling Density Maps

Each voxel of a cryo-EM density map has a density number whose value positively correlate with the possibility of the presence of electron of an atom in the voxel, which can be used as input features to predict positions and types of protein atoms. After a broad review [5] of the structural properties that current machine learning methods aim to predict from density maps, we created the following labels for voxels in a density map: atom labels, amino acid labels, and secondary structure labels by matching (fitting) the density map and its corresponding known protein structure.

For atoms labeling, we constructed a new mask map with the same dimension as the original density map that was initially filled with values of 0, indicating that the map was empty. We then utilized the corresponding protein structure for the density map to label each voxel containing backbone carbon-*α* atom (C*α*) as 1, backbone nitrogen atom (N) as 2, and another backbone carbon atom (C) as 3. The mask map created from the original unlabeled density map is a 3D grid, where the location of each voxel is determined by indices (i, j, k). But the corresponding protein structure used to label the voxels are in a 3D coordinate system (x, y, z). Therefore, we calculated the corresponding indices of each backbone atom (C*α*, N and C) in the mask map from its atomic coordinates using the Formula 1, where i, j, k are the grid indices of the atom in the mask map, x, y, z are the coordinates in the protein structure, $origin_{x}$, $origin_{y}$, $origin_{z}$ are the origin of x, y, z axis respectively found in the original experimental map’s metadata, and $voxel_{x}$, $voxel_{y}$, $voxel_{z}$ are the voxel size of x, y, z axis respectively found in experimental map’s metadata.

**Figure 3:**
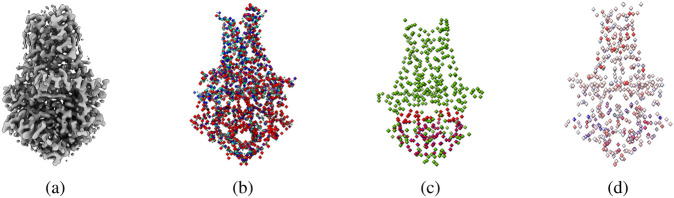
An example of labeling a cryo-EM density map. (**a**) The density map of SARS-CoV spike gycoprotein, EMD-22898 visualized at recommended contour level of 0.7. (**b**) Three different types of protein backbone atoms (C*α*, N, C) labeled in different colors. (**c**) Three different secondary structure elements labeled in different colors. (**d**) Twenty different amino acid labeled in different colors. The image are generated using UCSF ChimeraX’s [23] surface color by volume data value.

Similar to the atoms labeling, we created a mask map containing amino acid type labels. We labeled each voxel of containing a C*α* atom with one of 20 different types of standard amino acids, represented by 20 numbers from 1 to 20, while 0 denotes the absence of Ca atom or unknown amino acid type. Moreover, following the same approach used for atoms and amino acid types labeling, we created a mask map for secondary structure labels. We used UCSF ChimeraX [23] to identify and extract the secondary structure type (coil, *β*-strand, and *α*-helix) for each C*α* atom from the protein structure. The extracted secondary structures were then used to label each voxel of a 3D density map that contains a C*α* atom. We used 1, 2, and 3 to represent coil, *α*-helix, and *β*-strand, respectively. Figure 3 shows an example of density map labels.


(1) $$i=\lceil \left(\frac{\left\lfloor (z-origin_{z}\right)}{voxel_{z}}\right);j=\lceil \left(\frac{\left\lfloor (y-origin_{y}\right)}{voxel_{y}}\right);k=\lceil \left(\frac{\left\lfloor (x-origin_{x}\right)}{voxel_{x}}\right)$$

### 4.4 Data Splits

We selected 208 cryo-EM density maps that were used as test data in the previous works [14, 24] from our dataset as the test dataset. The remaining 7,392 density maps were split into the training dataset consisting of 6,652 maps and the validation dataset consisting of 740 maps according to 90% and 10% ratio. Table 1 reports the number / percentage of maps in the training and validation datsets for each resolution range, indicating the statistics for the two datasets is largely consistent. If necessary, users may choose split Cryo2Struct into training, validation and test datasets differently.

### 4.5 Grid Division

The dimensions of density maps of different proteins vary and are usually too large to fit into the memory of a standard GPU for training deep learning models. Similar to the approach employed in DeepTracer [14], Haruspex [15], CR-I-TASSER [18], Emap2sec [19], and Cascaded-CNN [9], we performed grid division to divide the density maps into 3D subgrids with dimension of 32 × 32 × 32 overlapped by 6 voxels on each face of the subgrid to train deep learning methods. We choose the dimension 32 as it is big enough to capture the patterns in the data and is small enough to be used with GPUs effectively. In the inference stage, the predictions for the sub-grids can be stitched back to obtain the prediction for the full density map by concatenating the predictions for the central 20 × 20 × 20 core voxels of the sub-grids. This approach allowed us to preserve the spatial information of the density maps and overcome the abnormal predictions for the voxels being cut off at boundaries of sub-grids during the grid division. Figure 2c shows an example of grid division. The sub-grids of each density map along with their corresponding labeled mask maps are saved as a single entity in numpy array for deep learning training and test. In total, there are ~ 39 million training sub-grids and ~ 4 million validation sub-grids. Moreover, the scripts of dividing density maps are provided at the Cryo2Struct’s GitHub repository for users to create sub-grids of their defined dimensions if necessary.

## 5 Validation of Cryo2Struct

To validate the utility and quality of Cryo2Struct, we designed two deep transformer models and trained and test them on Cryo2Struct to predict backbone atoms and amino acid types from density maps. Moreover, we used predicted C*α* atom positions and probabilities of 20 amino acid types to construct a Hidden Markov Model (HMM) model [25]. The HMM model was used to align protein sequences into predicted C*α* positions to build the backbone structures of proteins via a customized Viterbi [26] algorithm.

### 5.1 Training and Testing Deep Transformer Models on Cryo2Struct

#### Deep Learning Architecture

Inspired by the successful application of Transformer [27] in natural language processing (NLP), we formulated the task of classifying voxels in cryo-EM density maps as a sequence-to-sequence prediction problem. We utilized the UNEt TRansformers (UNETR) [28] architecture to translate density maps to their backbone atom or amino acid type labels, which includes a transformer block to directly process the embedded 3D cryo-EM maps, enabling it to capture long-range voxel-voxel dependencies. Additionally, a skip-connected decoder is employed to combine the extracted representations at different resolutions to predict the labels of voxels (backbone atom types or amino acid types).

#### Implementation

We trained two UNETR models from scratch on the density map sub-grids of 32 × 32 × 32 and a batch size of 720. We used the weighted cross entropy loss to compute loss between the input logits and target labels because voxels containing protein backbone atoms only account for a small portion of all the voxels. By assigning a higher weight to the pixels with positive labels, the models can pay sufficient attention to them. We trained the models using NADAM [29] optimizer with learning rate of 1e-4 and dropout of 0.1. The models were trained in distributed fashion on 24 compute nodes, each containing 6 NVIDIA V100 GPUs with 32 GB memory [30]. During the training, the training examples were shuffled, and the loss were tracked. If the loss did not improve for 5 consecutive epochs, the learning rate was reduced by a factor of 0.1.

One model was trained to classify each voxel into one of four different classes representing three backbone atoms (C*α*, C and N) and no presence of any backbone atoms. Another model was trained to classify each voxel into one of twenty-one different amino acid classes representing twenty different amino acids and unknown or absence of amino acid. In the inference stage, the predictions for all the sub-grids of a full density map are combined to generate a prediction for the entire map.

### 5.2 HMM-Guided Alignment of C*α* Atoms and Protein Sequences

Connecting the predicted carbon-*α* atoms into protein chains and determining their amino acid types accurately is one of the most challenging aspects of reconstructing protein structure from a cryo-EM density map. To address this challenge, we constructed a novel HMM from C*α* atoms and their amino acid types predicted by the deep transformer models. This model aligns the known protein sequences with the predicted the hidden states denoting C*α* atoms in the HMM, allowing us to link C*α* atoms into protein chains and assign amino acid types to the atoms in a single step.

Specifically, the predicted C*α* positions from the atom prediction are used as hidden states in the HMM, which are fully connected to generate the sequences of a protein. The amino acid emission probabilities of each C*α* hidden state are the geometric mean of the probabilities of twenty different amino acid types predicted by the amino acid type prediction model and their background probabilities precomputed from the protein sequences in the training data. The geometric mean is computed as $\sqrt{a\times b}$, where a and b represent the predicted probability for an amino acid type and its frequency, respectively.

The transition probability between C*α* hidden states are calculated according to the euclidean distance (x) between two C*α* atoms. The computed distance x is converted into the probability using the Gaussian probability density function in Formula 2. (f(x)) is computed with mean (μ) of 3.8047 and standard deviation (σ) of 0.036. Both the μ and σ for the distance between two C*α* atoms are precomputed from the known protein strucures in the training dataset.


(2) $$f(x)=\frac{1}{\sigma \sqrt{2\pi }}e^{-(x-\mu {)}^{2}∕2\sigma^{2}}$$

Because it is not known which C*α* state generates the first amino acid of a protein chain, the HMM allows every C*α* hidden state to be the initial state. The probability for a C*α* state to be the initial state is equal to the probability of it emitting the first amino acid divided by the sum of the probability of every state emitting the first amino acid. The constructed HMM is then used by a customized Viterbi algorithm to compute the most likely path of aligning protein sequences with the C*α* hidden states, resulting a determined protein backbone structure. The customized Viterbi algorithm allows any C*α* state to occur at most once in the path because one C*α* position can be occupied by only one amino acid of a protein.

### 5.3 Evaluation Results on SARS COVID-19 Proteins

**Figure 4:**
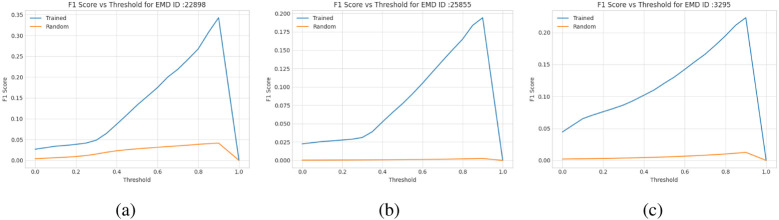
F1 scores of C*α* atom predictions for the density maps of two SARS COVID-19 (EMD-22898, EMD-25855) and a human p97 (EMD-3295) test proteins. The curve in blue denotes how F1 score of the deep transformer trained on Cryo2Struct changes with respect to the threshold on predicted Ca atom probabilities and the curve in orange the random predictions.

We tested the trained transformer models on three SARS-CoV-2 proteins [31, 32, 33] and one human p97 protein [34]. The voxel-wise predictions of C*α* atom were evaluated using F1-score (i.e., geometric mean of precision and recall of C*α* predictions). We used F1-score as it is a more balanced metric than the accuracy when there is a significant imbalance in the class distribution (i.e., the portion of voxels containing Ca atoms is very small in this case). We compare the F1 scores of our predictions with those of the random predictions in Figure 4. The former is much better the latter.

We further evaluated the C*α* backbones aligned by the HMM for the two COVID-19 proteins and the human p97 protein against the known protein structures. To adhere to the commonly practiced approach in the literature [14, 24], we used phenix.chain_comparison tool to compute the root mean squared distance (RMSD), the percentage of matching C*α* atoms, and the percentage of sequence identity. Phenix’s chain_comparison tool compares two structures to identify the number of matching C*α* atoms. Using this approach, it calculates the matching percentage, which represents the proportion of residues in the known structure that have corresponding residues in the reconstructed backbone structure. Similarly, it reports the sequence matching percentage indicating the percentage of matched residues that have the same amino acid type. The results in Table 2 shows that using the cryo2struct dataset with the transformer predictions and HMM alignment, we were able to determine the backbone structures of the four proteins with the good accuracy on average. Appendix A.1 shows several good, detailed examples of predicting Ca atoms and reconstructing protein backbone structures from density maps.

## 6 Conclusion

We created Cryo2Struct, a large, validated, high-quality dataset for the AI and bioinformatics community to develop AI methods for accurately and automatically reconstructing large protein structures from cryo-EM density maps. It fills a critical gap of lacking well-curated, publicly available cryo-EM density map datasets in the field to enable the development. The dataset of this scale allows researchers to train and test robust and powerful deep learning models to predict the positions of protein backbone atoms (e.g., C*α* atoms) and their amino acid types in 3D cryo-EM density maps, which can be linked together to build 3D protein structures from scratch without using any known structural information as templates. The AI-powered de novo reconstruction of protein structures from cryo-EM density maps will significantly extend the capability and efficiency of cryo-EM techniques to solve the structures of large protein complexes and assemblies.

## 7 Societal Impact

This dataset will enable AI scientists to develop AI technologies to automate the process of accurately reconstructing protein structures from cryo-EM data, which will have a significant impact on protein structure-based drug discovery and biotechnology development as well as renewable energy production, food production, disease treatment, and healthcare benefiting from the technologies. There is no negative societal impact noticed.

## 8 Limitations

There are different ways to normalize the density values of cryo-EM density maps. Cryo2Struct currently only supports one way based on 95% percentile. If other normalization methods are more effective for machine learning, they will be added in the future.

## Figures and Tables

**Table 1:** The resolution distribution of the maps in the training and validation datasets. Count refers to the number of cryo-EM density maps within a specified resolution range in a dataset.

	Train Set		Validation Set	
Resolution Range (Å)	Count	%	Count	%
1.0 - 2.0	62	0.93	5	0.67
2.0 - 3.0	2147	32.27	222	30.00
3.0 - 4.0	4443	66.79	513	69.32
Total	6652		740	

**Table 2:** Evaluation scores for predicted backbone structures for three SARS COVID-19 proteins one human p97 protein (EMD-3295)

EMDB	PDB	Residues	RMSD (↓)	Matching (%, ↑)	Sequence ID (%, ↑)
22898	7KJR	448	1.13	78.6	83.5
30210	7BV2	1036	1.31	69.7	73.0
25855	7TEY	2703	1.42	71.6	85.0
3295	5FTJ	4338	1.55	62.7	21.0
Average			1.35	70.65	65.62

**Figure 5:**
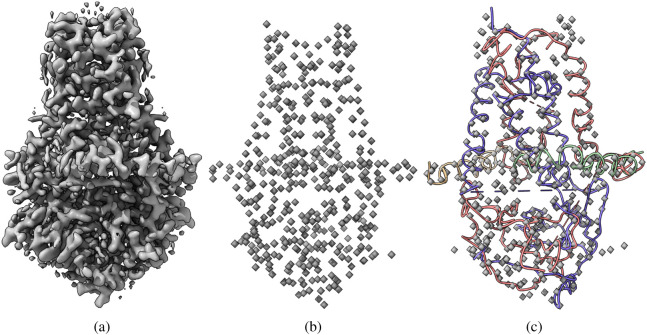
(a) The Cryo-EM density map of SARS-CoV-2 ORF3a (EMD-22898) visualized at the recommended contour level of 0.7 (10.3 σ). The map dimension is 300 × 300 × 300 and has density values between −2.319 – 3.909. The voxel dimensions is 0.727 × 0.727 × 0.727 (Å). (b) The true C*α* atom voxels extracted from the density map. (c) The aligned C*α* backbone structure using HMM, overlaying with true C*α* voxels.

**Figure 6:**
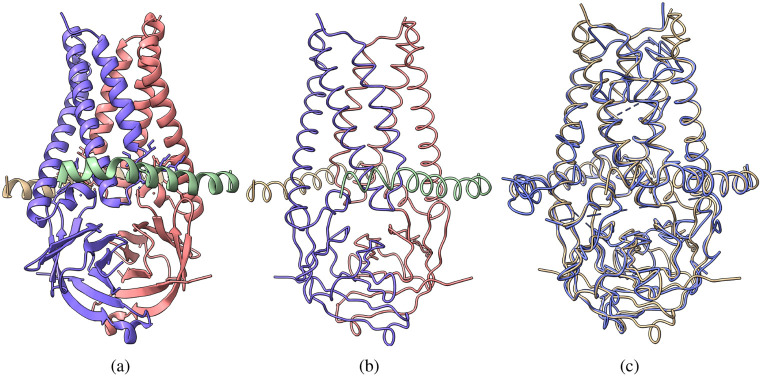
(a) The known protein structure corresponding to the density map EMD-22898 (PDB code 7KJR). The known structure has 448 residues. (b) The true C*α* backbone structure extracted from the known PDB structure. (c) The superimposition of the predicted backbone (blue) structure with the known backbone structure (gold).

**Figure 7:**
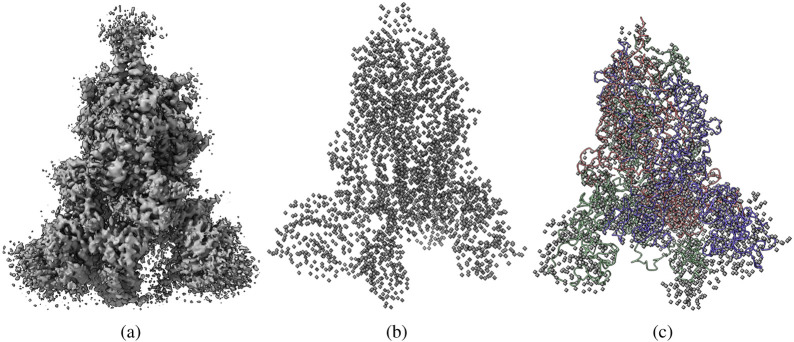
(a) The Cryo-EM map of SARS-CoV-2 Delta (B.1.617.2) spike protein (EMD-25855) visualized at recommended contour level of 0.121 (5.0 σ). The map dimension is 400 × 400 × 400 and has density values between −0.467 – 1.315. The voxel dimensions is 1 × 1 × 1 (Å). (b) The true C*α* atom voxels extracted from the density map. (c) The aligned C*α* backbone structure using HMM, overlaying with true C*α* voxels.

**Figure 8:**
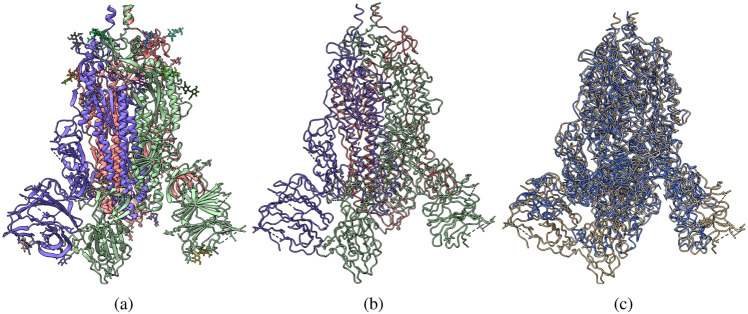
(a) The known protein structure of the density map EMD-25855 (PDB code 7TEY). The known structure has 2,703 residues. (b) The true C*α* backbone structure extracted from the known protein structure. (c) The superimposition of the predicted backbone structure (blue) with the known backbone structure (gold).

**Figure 9:**
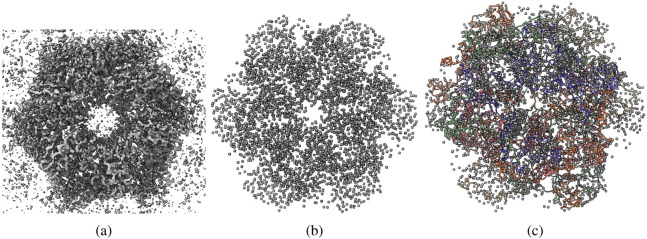
(a) The Cryo-EM density map of human p97 bound to UPCDC30245 inhibitor (EMD-3295) visualized at recommended contour level of 0.0375 (3.4 σ). The map dimension is 312 × 312 × 312 and has density values between 0.085 – 0.118. The voxel dimensions is 0.637 × 0.637 × 0.637 (Å). (b) The true C*α* atom voxels extracted from the deposited density map. (c) The aligned C*α* backbone structure using HMM, overlaying with the true C*α* voxels.

**Figure 10:**
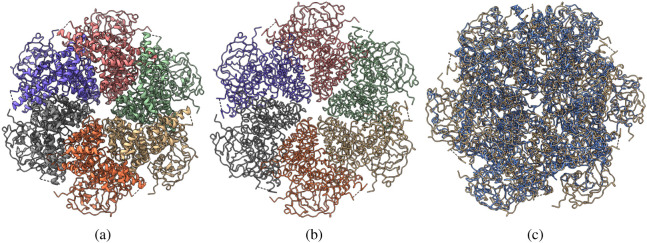
(a) The known protein structure of the density map EMD-3295 (PDB code 5FTJ). The structure has 4,338 residues. (b) The true C*α* atom backbone structure extracted from the known protein structure. (c) The superimposition of the predicted backbone structure (blue) with the known backbone structure (gold).

## Checklist

1. For all authors…
  - Do the main claims made in the abstract and introduction accurately reflect the paper’s contributions and scope? [Yes]
  - Did you describe the limitations of your work? [Yes] See Section 8
  - Did you discuss any potential negative societal impacts of your work? [Yes] See Section 7
  - Have you read the ethics review guidelines and ensured that your paper conforms to them? [Yes]
2. If you are including theoretical results…
  - Did you state the full set of assumptions of all theoretical results? [N/A]
  - Did you include complete proofs of all theoretical results? [N/A]
3. If you ran experiments (e.g. for benchmarks)…
  - Did you include the code, data, and instructions needed to reproduce the main experimental results (either in the supplemental material or as a URL)? [Yes] We provide a GitHub URL : https://github.com/BioinfoMachineLearning/cryo2struct.
  - Did you specify all the training details (e.g., data splits, hyperparameters, how they were chosen)? [Yes] See Section 5.1 ans 4.4
  - Did you report error bars (e.g., with respect to the random seed after running experiments multiple times)? [N/A]
  - Did you include the total amount of compute and the type of resources used (e.g., type of GPUs, internal cluster, or cloud provider)? [Yes] See Section 5.1 and 9
4. If you are using existing assets (e.g., code, data, models) or curating/releasing new assets…
  - If your work uses existing assets, did you cite the creators? [Yes] We have cited both Electron Microscopy Database (EMDB) and Protein Data Bank (PDB).
  - Did you mention the license of the assets? [Yes] PDB and EMDB have license - CC0. Data files contained in the EMDB and PDB are free of all copyright restrictions and made fully and freely available for both non-commercial and commercial use. For further information: https://www.ebi.ac.uk/emdb/documentation/faq (EMDB policies) and https://www.wwpdb.org/about/usage-policies (PDB policies)
  - Did you include any new assets either in the supplemental material or as a URL? [Yes] We provide a GitHub URL : https://github.com/BioinfoMachineLearning/cryo2struct.
  (d) Did you discuss whether and how consent was obtained from people whose data you’re using/curating? [N/A]
  (e) Did you discuss whether the data you are using/curating contains personally identifiable information or offensive content? [N/A]
5. If you used crowdsourcing or conducted research with human subjects…
  - Did you include the full text of instructions given to participants and screenshots, if applicable? [N/A]
  - Did you describe any potential participant risks, with links to Institutional Review Board (IRB) approvals, if applicable? [N/A]
  - Did you include the estimated hourly wage paid to participants and the total amount spent on participant compensation? [N/A]

## A Appendix

### A.1 Several examples of predicting Ca atoms and reconstructing protein backbone structures

Figures 5, 7, 9 visualize and analyze the predicted Ca backbone structures of three proteins with respect to the true Ca atoms of the density maps. The predicted backbone structures were constructed by the deep transformer trained on Cryo2Struct and HMM. Figures 6, 8, 10 compare the predicted backbone structures of the same three proteins with their true backbone structures extracted from the known protein structures. We used UCSF ChimeraX [23] for the visualization of the cryo-EM density maps and the protein structures. Generally, the reconstructed backbone structures match the true Ca atoms in the density maps and the true backbone structures reasonably well.
